# Printable Fluorescent Hydrogels Based on Self-Assembling Peptides

**DOI:** 10.1038/s41598-017-10162-y

**Published:** 2017-08-29

**Authors:** Yifan Xia, Bin Xue, Meng Qin, Yi Cao, Ying Li, Wei Wang

**Affiliations:** 1 National Laboratory of Solid State Microstructures, Department of Physics, Nanjing University, 22 Hankou Road, Nanjing, Jiangsu 210093 P.R. China; 2 Collaborative Innovation Center of Atmospheric Environment and Equipment Technology, Jiangsu Engineering Technology Research Centre of Environmental Cleaning Materials, Jiangsu Key Laboratory of Atmospheric Environment Monitoring and Pollution Control, Jiangsu Joint Laboratory of Atmospheric Pollution Control, Jiangsu School of Environmental Science and Engineering, Nanjing University of Information Science & Technology, 219 Ningliu Road, Nanjing, Jiangsu 210044 P.R. China

## Abstract

Fluorescent hydrogels (FH) have a variety of potential applications in the field of soft electronics. However, fabrication of mechanically stable and printable fluorescent hydrogels remains challenging. Here, we report a kind of fluorescent hydrogel based on the co-assembly of peptide motif and transition metal ions. The metal ions are captured in the hydrogel network at specific positions through covalently linked ligands on the peptide hydrogelators. This efficiently prevents the aggregation and self-quenching of organometallic chromophores. In addition, the formation of metal-ligand complexes introduces additional interactions to stabilize the hydrogel network, making the FH even more stable after the incorporation of metal ions. The FH is optically transparent but highly fluorescent. By using three different metal ions, the white light fluorescent supramolecular hydrogel has been achieved. As a proof-of-principle, we demonstrate the printability of the hydrogels to various patterns. We anticipate that with the improved fluorescent performance and stability, this kind of FH can find broad applications in extrusion-based 3D printing for the construction of soft electronics.

## Introduction

Light-emitting materials have attracted significant attentions because of their broad potential applications^1^, such as light-emitting diodes^2–5^, displays^5–7^, photonics^8^, sensors^9, 10^, artificial photosynthesis^11, 12^, solar cell devices^13–15^ *etc*. Especially in the field of the white light-emitting materials, an increasing need for the moldable solid white light source has appeared. Usually, the materials for generation of white light emission are based on inorganic complexes^16^, metal–organic frameworks^17, 18^, quantum dots^19^, organic molecules^20–24^, and nanomaterials^25, 26^.

Among the light-emitting materials, white-luminescent solid materials based on supramolecular design have been extensively investigated because the organization of donors and acceptors can be easily modulated by rationally designing the self-assembled supramolecular structures of the nanomaterials, such as hydrogel^23, 27–30^, nanofibres^31–34^, nanoparticles^28, 35–37^, and etc^38–40^. In particular, hydrogels^41–47^, known as soft yet moldable materials, are suitable to construct solid fluorescent light-emitting devices due to the convenient synthetic procedures and preparation methods. Fluorescent hydrogels (FH) can be readily prepared by introducing various chromophores of organometallic complexes or fluorescent dyes into the hydrogels. The photo-luminescent properties of dyes can be specifically enhanced and tuned by the unique nanostructures and chemical environments of the hydrogels^48–50^. Moreover, by rational design, it is possible to further improve the fluorescent properties of FH by rigidifying the donor−acceptor pairs and reducing the nonradiative decay. Another advantage of FH, especially the supramolecular ones, is their unique physical properties: They are often elastic, injectable and healable after damage. They can even function in water, which is appropriate for artificial underwater robots^24, 51, 52^.

However, most FH reported so far were constructed by simply physically mixing the fluorescent dyes with hydrogelators or soaking preformed hydrogels in solutions containing fluorescent molecules^53, 54^. Lack of direct chemical bonding of the dye molecules with the hydrogel network may cause the leaking of the fluorescent materials as well as the aggregation of the chromophores, leading to fluorescence quenching and reduction of luminescence^55, 56^. Moreover, the introduction of chromophores may also affect the chemical and mechanical stabilities of the hydrogels, in that most chromophores are bulky and hydrophobic, which may destabilize the hydrogel network. It remains a significant challenge to generate bright and stable FH for practical applications.

In this work, we have designed a novel FH based on the co-assembly of metal-ligand complexes with peptide hydrogelators in a single step. The ligands were covalently linked to the peptide hydrogelator. Such a design is kind of “killing two birds with one stone”. First, because the metal ions are fixed to the hydrogel network at specific positions, the aggregation and self-quenching of chromophores are prevented. Second, the formation of metal-ligand complexes could introduce additional interactions to stabilize hydrogel network, making the FH even more stable after the incorporation of metal ions. The structural, physical and optical properties of the hydrogels were studied in details. This type of FH based on self-assembled peptides shows high transmittance, color-switchable luminescence and great stability in water. Moreover, by using three different metal ions, the white light fluorescent supramolecular hydrogel has been achieved with Commission Internationale de L’Eclairage (CIE) coordinates of (0.33, 0.33). As a proof-of-principle, we demonstrated the printability of the hydrogels to various patterns functioning in aqueous conditions. We anticipate that with the improved fluorescent performance and stability, this kind of FH can find broad applications in 3D printable soft electronics.

## Results Section

### Design of the fluorescent hydrogel

**Figure 1:**
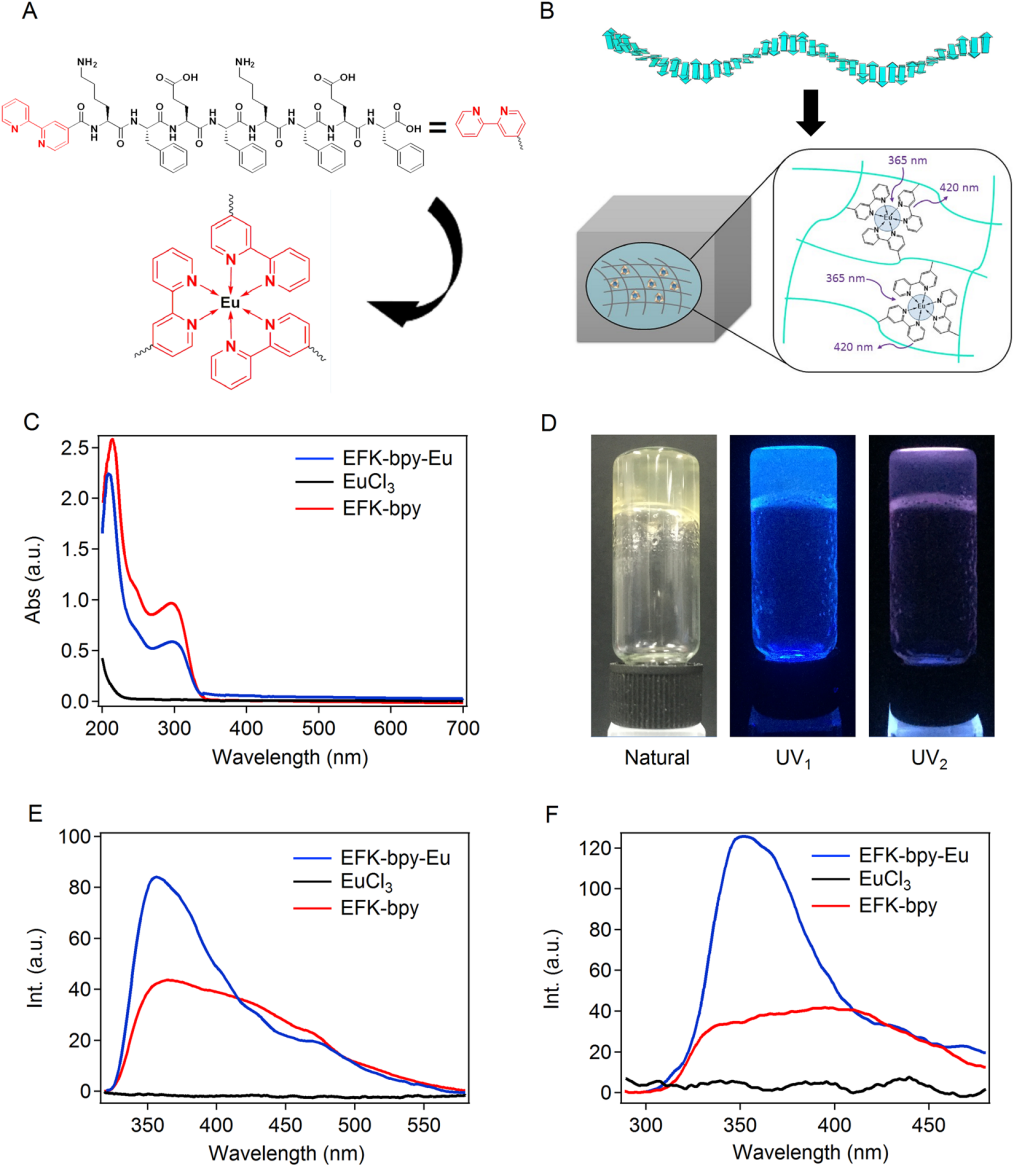
Schematic illustration and fluorescence of the self-assembly peptides. (**A**) Molecular structures of the EFK-bpy peptide and the structures of the EFK-bpy-Eu peptide. (**B**) Proposed schematic representation of the co-assembled hydrogel and the energy-transfer process. (**C**) The UV-Vis absorption spectra of the EFK-bpy hydrogel (4 mM), EuCl_3_ (1.3 mM), and EFK-bpy-Eu hydrogel (1.3 mM). (**D**) Optical images of the EFK-bpy-Eu hydrogel under natural light (left), UV_1_ (middle, 330–390 nm) and UV_2_ (right, 220–280 nm) irradiation from the handheld UV lamp. (**E**) The fluorescence emission spectrum of the EFK-bpy hydrogel (λ_ex_ = 300 nm; 4 mM), EuCl_3_ (λ_ex_ = 300 nm; 1.33 mM) solution, and EFK-bpy-Eu hydrogel (λ_ex_ = 300 nm; 1.33 mM). (**F**) The fluorescence emission spectrum of the EFK-bpy hydrogel (λ_ex_ = 254 nm; 4 mM), EuCl_3_ (λ_ex_ = 254 nm; 1.33 mM) solution, and EFK-bpy-Eu hydrogel (λ_ex_ = 254 nm; 1.33 mM).

Many short peptides are well known for their ability to self-assemble into fibrous network structures and form supramolecular hydrogels. Among these peptides, ionic-complementary peptides were widely reported to form hydrogels by self-assembling into long β-sheet fibres^57–60^. As shown in Fig. 1A, the ionic complementary peptide sequence KFEFKFEF was chosen as the self-assembly motif for the White light fluorescent hydrogel^61–65^. Ionic complementary peptides are well known for its robust self-assembly properties. As far as the specific charge periodicity in the sequence is maintained, the chirality and sequence alternation do not affect the mechanical properties of the hydorgels^66^ (Figure S1). The N-terminus of the peptide was capped with the powerful ligand, 2,2′-bipyridine (bpy), for metal ion chelation. The final modified peptide was named as bpy-KFEFKFEF hereafter. Europium ion (Eu^3+^) was chosen as the metal ion center because of its outstanding fluorescent property. Moreover, it can chelate with three bpy ligands to form an octahedral complex (Fig. 1A). Introducing bpy did not affect the self-assembly of KFEFKFEF. Bpy-KFEFKFEF can still self-assemble into fibrous hydrogel structures at neutral pH due to the ionic interactions between positively charged protonated amino groups on the side chain of lysine (K) and negatively charged carboxylate group on the side chain of glutamic acid (E) as well as the hydrophobic interactions and π-π stacking interaction among phenylalanine residues (Fig. 1B). The addition of Eu^3+^ could crosslink the peptide fibres through metal-ligand coordination (Fig. 1B)^63^. The hydrogels with and without Eu^3+^ ions are named as EFK-bpy-Eu hydrogel and EFK-bpy hydrogel, respectively.

### Optical properties of FH

The UV-Vis spectra of EFK-bpy-Eu hydrogel were measured to investigate the light absorption properties. For comparison, the UV-vis spectra of EuCl_3_ solution and EFK-bpy hydrogel were also measured (Fig. 1C). EuCl_3_ only exhibits weak absorption between the wavelengths of 200–400 nm. However, the EFK-bpy shows strong absorption in this region with three peaks locating at 222, 250 and 300 nm, respectively. The first two peaks can be attributed to the π-π* transition of phenyl groups of the peptide and the later one corresponds to the π-π* transition of bpy^67^. The presence of Eu^3+^ in the hydrogel lowers the intensities of these peaks but does not affect their positions. The hydrogel shows no optical absorption in the visible light region, indicating that it is transparent. In addition, the photo-luminescent properties of the hydrogel were also studied. The images of the EFK-bpy-Eu hydrogel under natural light, and two UV ranges (UV_1_: 330–390 nm and UV_2_: 220–280 nm) from the handheld UV lamp are shown in Fig. 1D, demonstrating the fluorescent properties of the hydrogel under UV excitation. Fluorescence spectra indicate that the strong fluorescent property of the EFK-bpy-Eu hydrogel is originated from Eu^3+^-bpy binding: There is a blue fluorescent peak at 420 nm when EFK-bpy-Eu is excited at 300 nm, while neither EFK-bpy nor EuCl_3_ is fluorescent (Fig. 1E). The fluorescence emission peak of EFK-bpy does not affect the emission color of the hydrogel since 80% of the peak was not in the visible range. Moreover, the emission wavelength of the hydrogel is switchable, depending on the excitation wavelength (Fig. 1F).

### Mechanical properties of FH

**Figure 2:**
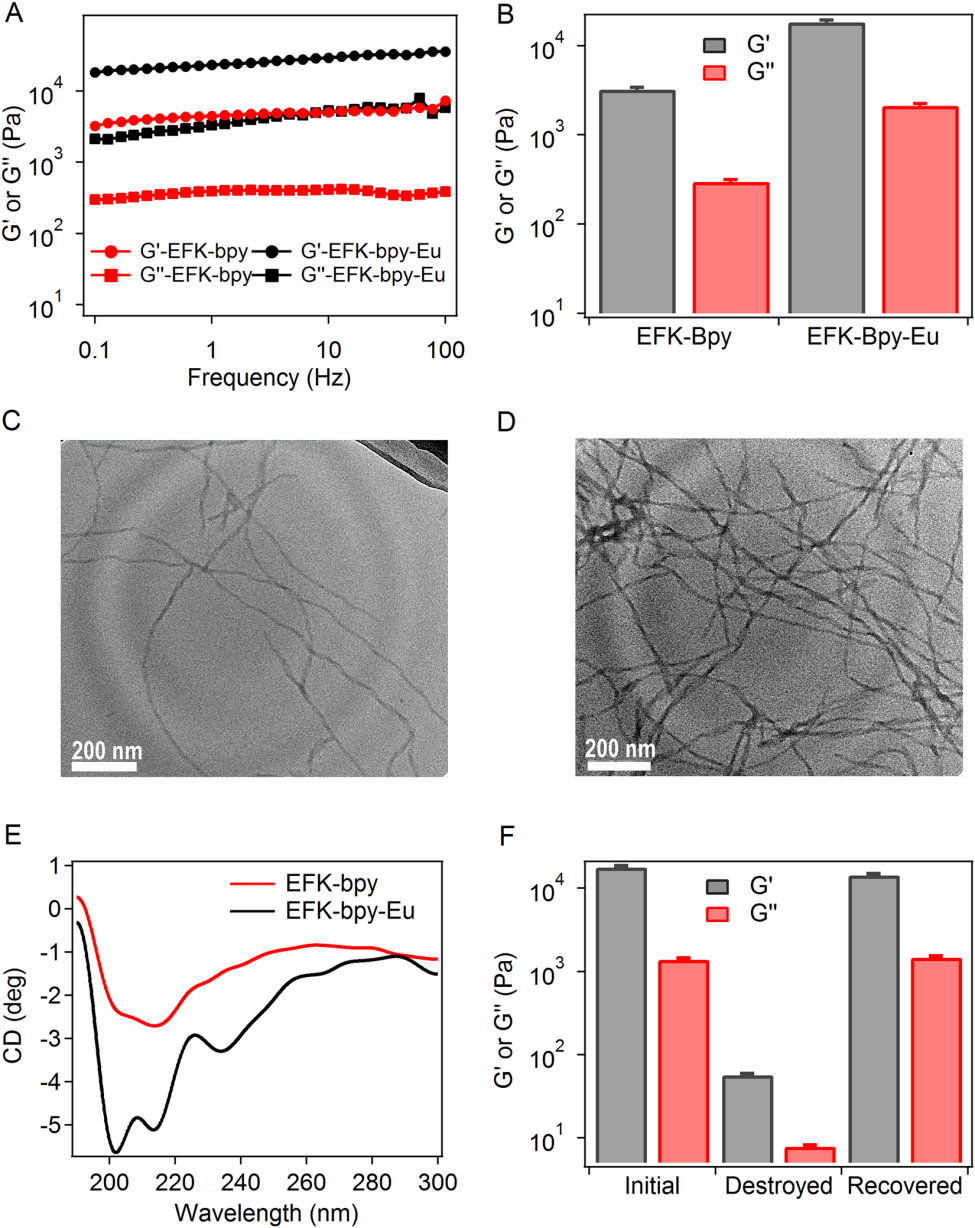
Mechanical and recovery properties of the hydrogels. (**A**) The rheological properties of EFK-bpy (12 mM) and EFK-bpy-Eu (4 mM) hydrogels at 0.1% strain in the frequency range of 0~100 Hz. (**B**) The G′ and G″ of EFK-bpy (12 mM) and EFK-bpy-Eu (4 mM) hydrogels at 1 Hz and 0.1% strain. (**C**) TEM images about the s entangled fibrous network of the EFK-bpy hydrogel. (**D**) TEM images about the s entangled fibrous network of the EFK-bpy-Eu hydrogel. (**E**) CD spectra of the EFK-bpy hydrogel (0.24 mM), and EFK-bpy-Eu hydrogel (0.08 mM). (**F**) The G′ and G″ of EFK-bpy-Eu hydrogels (4 mM) before damage (initial), right after damage (destroyed), and 2 h after damage (restored).

The mechanical properties of hydrogels are important for the applications as solid fluorescent gels. As illustrated in Fig. 2A, the storage modulus (G′) of the EFK-bpy-Eu hydrogel is about 20 kPa, more than 10 times higher than the loss modulus (G″) over a broad range of angular frequencies from 1 to 100 Hz. This is a clear evidence of the solid rather than viscous response of the gel. Additionally, the G′ of EFK-bpy-Eu hydrogel is about 6 times larger than that of the EFK-bpy hydrogel, implying that the increased crosslinking density through Eu^3+^-bpy coordination strengthens the mechanical property of the hydrogel (Fig. 2B). The increased crosslinking density is also evidenced at the microscopic level by TEM imaging (Fig. 2C and D). With Eu^3+^ ions, the peptide fibres in the hydrogel are thicker and more entangled due to the metal-ligand crosslinking. Eu^3+^-bpy binding also causes enhanced circular dichroism (CD) absorption of the hydrogel in the near-UV region, indicating that the secondary structure of the peptide fibres is stabilized in the EFK-bpy-Eu hydrogel (Fig. 2E). Because the hydrogel is stabilized by non-covalent interaction, it can recover its mechanical stability after damage. As displayed in Fig. 2F and S1, the initial hydrogel was destroyed by ultrasound for 30 min and then allowed to recover at room temperature for 2 h. After applying ultrasound treatment, the G’ reduced significantly from 2 × 10^4^ Pa to 80 Pa. However, it can be fully recovered after self-healing for 2 h. To understand the change in the mechanical properties by ultrasound at the microscopic level, TEM images of the initial, destroyed and recovered hydrogels were also studied. As shown in Figure S2 and Figure S9, the long fibers of the hydrogel were broken into shorter ones under sonication and the hydrogel turned to liquid. However, in the recovered hydrogel, the short fibers again grew into longer ones, suggesting that the self-assembly of the peptide hydrogelators is dynamic and reversible. We expect such a mechanical feature of the hydrogel making it a suitable “ink” for extrusion-based bioprinting.

### White light fluorescent hydrogel (WLFH)

**Figure 3:**
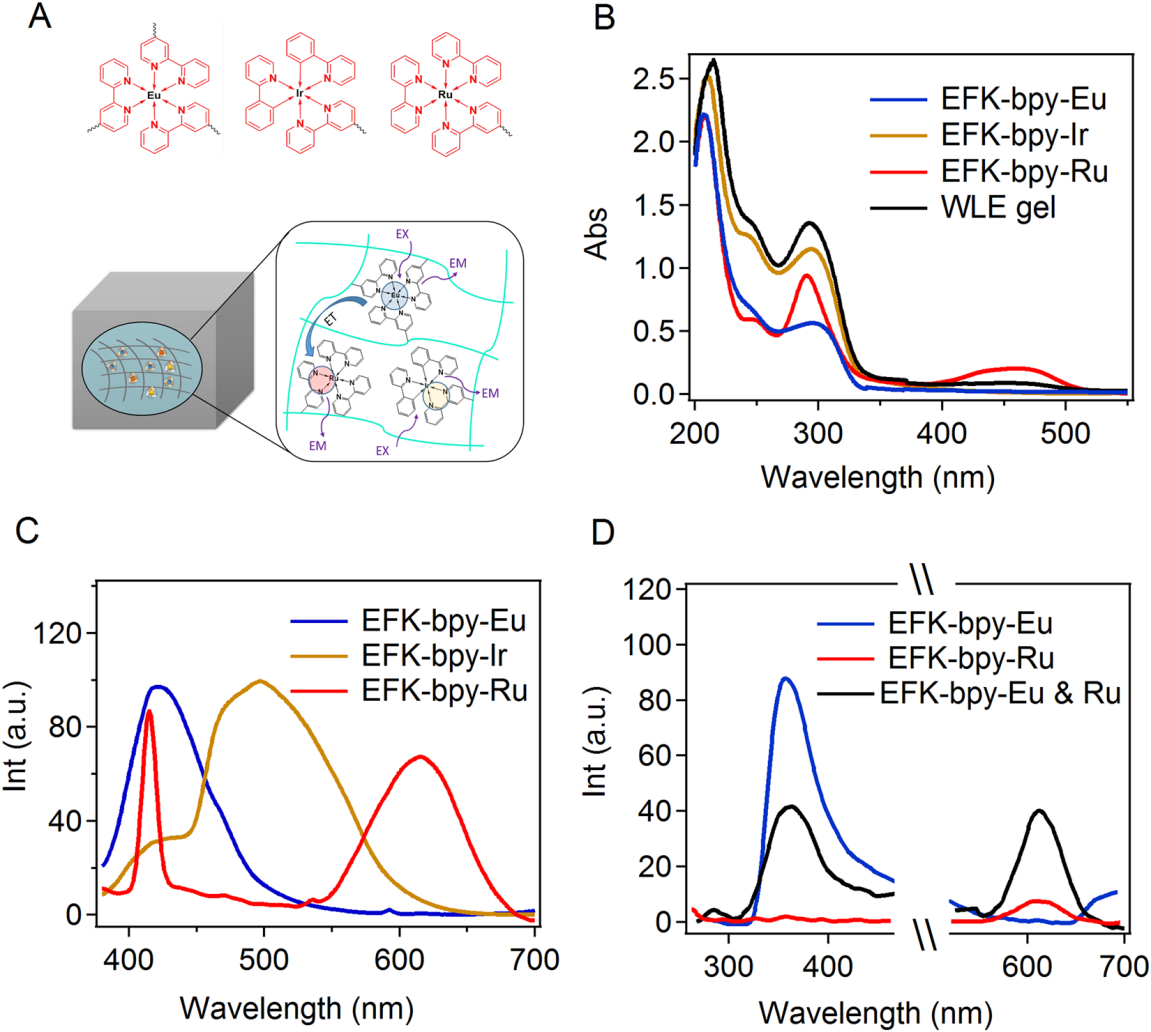
(**A**) Proposed schematic representation of the co-assembled hydrogel and the energy-transfer process. (**B**) The UV-Vis absorption spectra of the EFK-bpy-Eu (1.33 mM), EFK-bpy-Ir (4 mM), EFK-bpy-Ru (4 mM) hydrogels, and WLFH (4 mM). (**C**) The fluorescence emission spectrum of the EFK-bpy-Eu (λ_ex_ = 365 nm; 1.33 mM), EFK-bpy-Ir (λ_ex_ = 365 nm; 4 mM), and EFK-bpy-Ru (λ_exc_ = 365 nm; 4 mM) hydrogels. (**D**) The Förster resonance energy transfer between EFK-bpy-Eu (λ_ex_ = 254 nm; 1.33 mM) and EFK-bpy-Ru (λ_ex_ = 254 nm; 4 mM). The concentration of EFK-bpy-Eu and EFK-bpy-Ru in the mixture were 0.66 mM and 2 mM, respectively.

Among all the fluorescent materials, white ones are in great demand due to their potential applications in large-area flexible displays. Since 2,2′-bipyridine can form the organometallic complex with various ions, chromophores with different emitting light can be introduced into the hydrogel through the same coordination interactions. As shown in Fig. 3A, Eu^3+^, Ru^2+^, and Ir^2+^ were chosen to form the fluorescent metal complex with EFK-bpy peptide and prepared the WLFH, because their emission spectra are complementary in the visible light range. The chemical structures of the three different ionic compounds used to form ionic complexes were shown in Figure S3. The hydrogels with Ir^2+^ and Ru^2+^ ions can be prepared using the same procedure for EFK-bpy-Eu hydrogel (Figure S4) and are denoted as EFK-bpy-Ir hydrogel and EFK-bpy-Ru hydrogel, respectively, hereafter. They both show entangled fibrous network similar to the EFK-bpy-Eu hydrogel (Figure S5). The mechanical properties of these hydrogels are summarized in Figure S6 and are all stronger than that of EFK-bpy hydrogels^68–70^.

**Figure 4:**
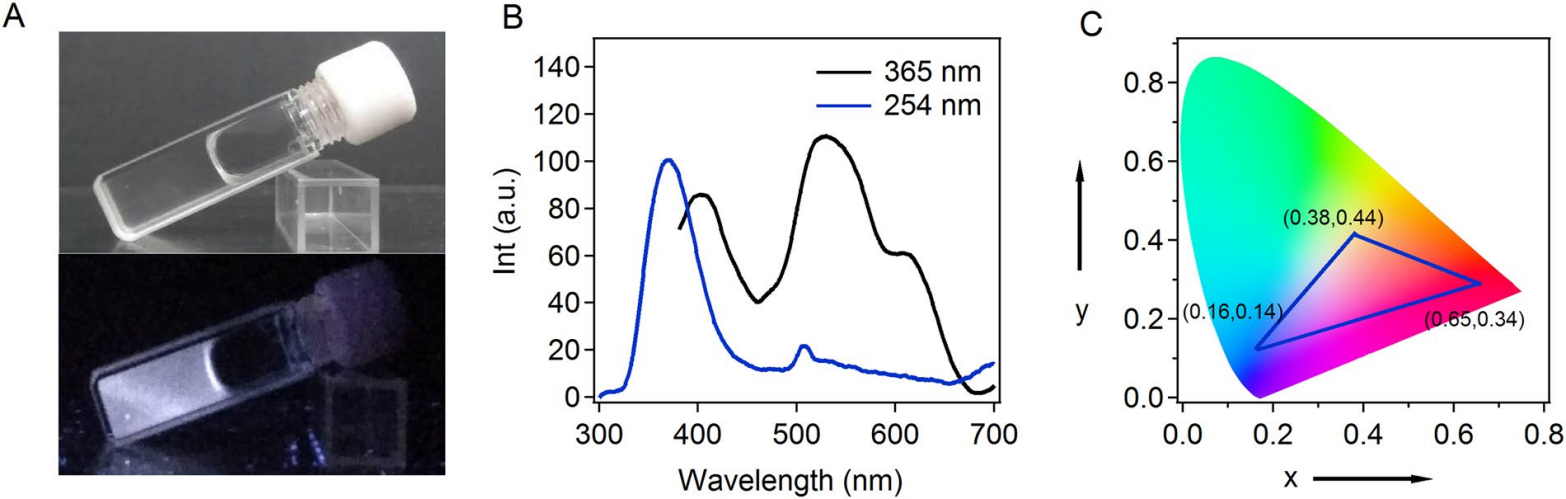
(**A**) The photographs of the WLFH (12 mM) under the illumination of natural light (top) and of UV_1_ (bottom). (**B**) The emission spectra of the WLFH (λ_ex_ = 365 nm and λ_ex_ = 254 nm). (**C**) CIE 1931 chromaticity diagram of the EFK-bpy-Eu, EFK-bpy-Ir, EFK-bpy-Ru hydrogels.

We then tuned the hydrogel composition for white light emission. We first characterized the UV-Vis absorption spectra of the hydrogels with only a single ligand (Fig. 3B). Obvious they all show strong absorption in the UV region. The emission spectra of the EFK-bpy-Eu, EFK-bpy-Ir and EFK-bpy-Ru hydrogels excited at 365 nm were shown in Fig. 3C. The emission spectra can cover the full visible light range, suggesting that it is possible to use these three components to construct white light fluorescent hydrogel. The EFK-bpy-Eu hydrogel exhibits blue emission at ~420 nm with CIE coordinates of (0.16, 0.14), the EFK-bpy-Ir hydrogel exhibits yellow emission at ~500 nm with CIE coordinates of (0.38, 0.44), and the EFK-bpy-Ru hydrogel mainly shows red emission at ~600 nm with CIE coordinates of (0.16, 0.14) even though there is a minor peak at 416 nm. Moreover, we found that there was Förster resonance energy transfer (FRET) in the EFK-bpy-Eu and EFK-bpy-Ru co-hydrogel system (Fig. 3D). This is reasonable because the emission spectrum of EFK-bpy-Eu overlaps with the excitation spectrum of EFK-bpy-Ru. However, no obvious FRET can be observed in the EFK-bpy-Eu and EFK-bpy-Ir co-hydrogel system as well as EFK-bpy-Ir and EFK-bpy-Ru co-hydrogel system (Figure S7). Due to the complicated photophysical processes in the hydrogel, the proper metal ion concentrations for white-fluorescent can only be obtained in a trial-and-error fashion. Luckily, we found that the peptide hydrogel containing Eu^3+^, Ir^2+^ and Ru^2+^ in a ratio of 20:1:0.5 can emit white light when being excited under the irradiation of UV_1_ lamp (Fig. 4A). The WLFH excited at 365 nm has three maxima emission wavelengths at 420 nm, 500 nm, and 600 nm, corresponding to blue, yellow and red parts of the electromagnetic spectra (Fig. 4B). The CIE value of (0.33, 0.33) for white fluorescent light can be achieved in this hydrogel. With different ratios of the three metal ions, hydrogels with a broad colour gamut could be achieved (Fig. 4C). Moreover, the range of the fluorescence emission light can be adjusted by using different excitation wavelengths (Figure S8).

### Potential applications of WLFH in aqueous conditions

**Figure 5:**
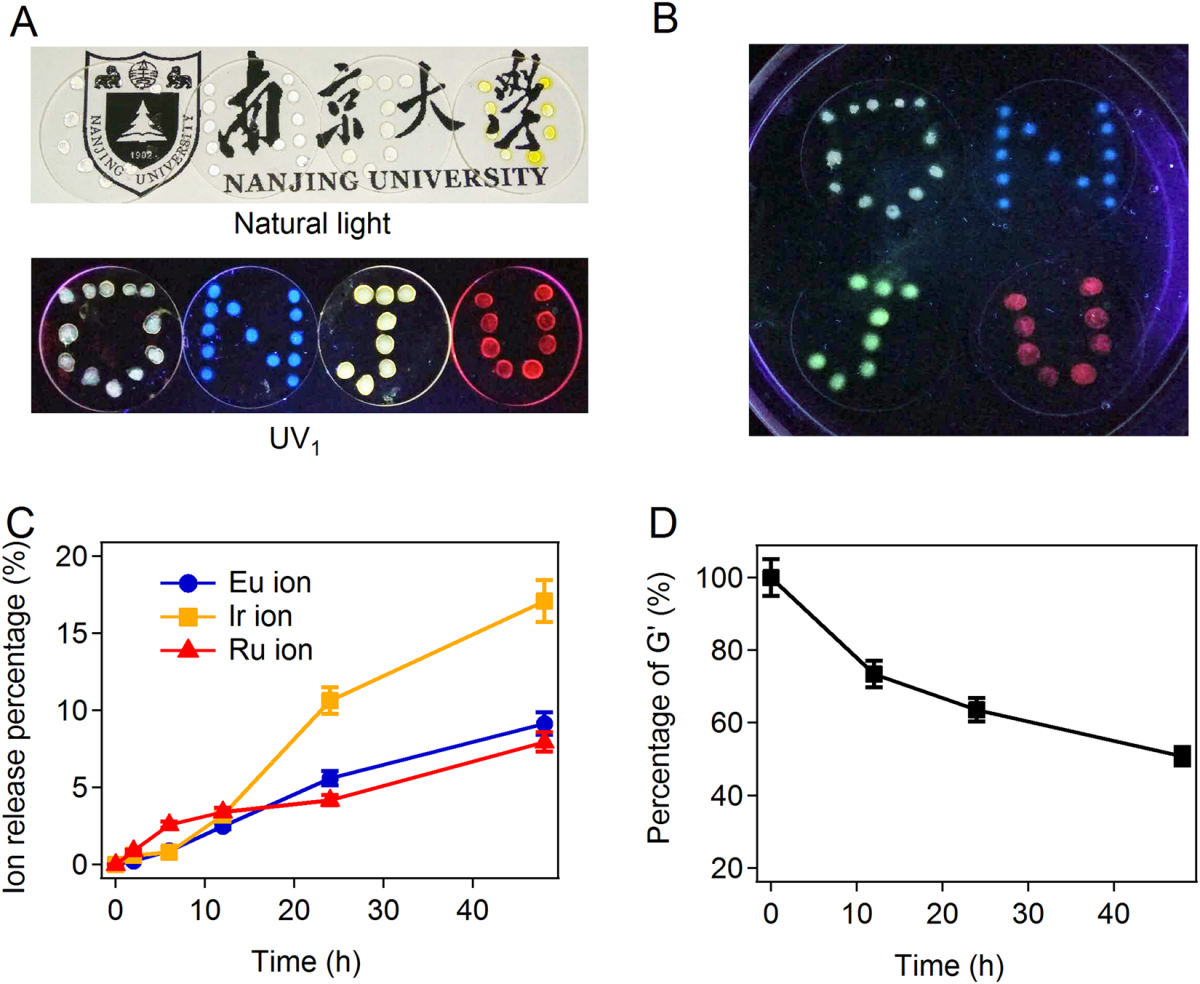
The printability and stability of WLFH. (**A**) The gels printed on quartz sheets. The gels remain transparent after the printing and show different light emission under the UV_1_ irradiation. (**B**) The hydrogels remain the original shapes and fluorescent properties in water for 4 h. (**C**) The ion release from the WLFH (12 mM) in a time period of 48 h in deionized water. (**D**) The normalized mechanical properties after 48-h incubation in deionized water compared to the original WLFH (12 mM).

A unique advantage of the supramolecular hydrogel is that their quick self-healing properties. The hydrogel can fully recover its mechanical stability after damage (Figure S9), suggesting that it can be used for extrusion-based 3D printing applications. A proof-of-principle demonstration of manually printing the hydrogels on silica glass surface by syringes was shown in Fig. 5. The stable hydrogel spots can form after being injected out of the syringes. These hydrogel spots were hard to be visualised by eyes because they are highly transparent. However, all the patterns showed excellent emission under the excitation of UV_1_ (Fig. 5A), illustrating the feasibility of using these hydrogels for fluorescent. Moreover, the hydrogels also functioned properly in water with low erosion rate because of the hyper-crosslinked network structure of the hydrogel (Fig. 5B). The hydrogel could retain its shape and fluorescent properties even after being placed in water for hours. The metal ion released into water was monitored by UV-vis spectroscopy. The released percentage of all the ions is less than 15% after soaking the hydrogel in water for 48 hours (Fig. 5C). The mechanical stability of the hydrogel decreases slightly after the incubation in water (Fig. 5D), which is probably due to the loss of EFK-bpy molecules. The photo stability of fluorescent gels is also excellent. The peak locations and intensities of EFK-bpy-Eu, EFK-bpy-Ir, and EFK-bpy-Ru hydrogels remained almost unchanged after storing in centrifuge tubes and illuminated under the natural light for 7 days (Figure S10). Even being soaked in water for 48 hours, all hydrogels remained fluorescent with some loss in fluorescence intensity (~20% for EFK-bpy-Eu and EFK-bpy-Ru hydrogels and ~38% for EFK-bpy-Ir hydrogel) (Figure S10). All these demonstrate the possibility of applying the hydrogel as photo-excitable underwater soft electronics.

## Discussion Section

In this work, we have demonstrated the fabrication of White light fluorescent hydrogel based on self-assembling peptides. There are several merits of this new type of fluorescent hydrogel that is worth of further discussion. First, the hydrogel is made of merely non-covalent interactions yet shows remarkable mechanical and chemical stability, thanks to the hyper-crosslinked structure. Both intra-fibre interactions (ionic interaction, hydrophobic interaction and π-π stacking) and inter-fibre interactions (metal-ligand coordination) are reversible. Therefore, the hydrogel can reform after being mechanically destroyed, making it a suitable candidate for 3D printing. Second, the hydrogel shows remarkable chemical stability and low erosion rate. Weak non-covalent interactions are typically dynamic. Therefore, most short-peptide based hydrogels tend to erode when being placed in water. However, with the help of additional metal-ligand bonds, the structure become much more stable and can function in aqueous conditions for several hours. Third, the hydrogel can display a wide range of color when using different transition metal centers. Because bpy is a common ligand for various metal ions with distinct luminescence, the emission color of the hydrogel can be easily tuned using different metal-ligand chromophores. Moreover, the introduction of ligands to the hydrogel network makes the hybrid system of high photo-stability by suppressing the aggregation and diffusion of chromophores. Fourth, the emission color of the hydrogel is switchable by changing the photo-excitation wavelength. The white-fluorescent can be achieved with a broad color gamut. Therefore, it may be used as a unique photo-controlled light source for special displays. Combining transition metal ions with self-assembling peptide in hydrogels may represent a general route to engineer hydrogel-based white-light-emitters.

## Conclusion

In summary, we report a new kind of fluorescent hydrogel based on self-assembly peptides hyper-crosslinked by coordination bonds. The hydrogel shows improved mechanical and chemical stability, broad colour gamut, switchable photo-induced emission spectra, and white light fluorescent properties. Moreover, it is printable and can be easily integrated into complicated electronic devices. The fluorescent properties and stability can be further tailored for different applications. We anticipate that it may find wide applications in the field of soft electronics.

## Methods Section

### Synthesis of peptides

The EFK-bpy peptide was bought from GL biochem (Shanghai) Ltd. The hydrogel based on ruthenium was prepared by mixing the EFK-bpy peptide (12 mM) with cis-bis(2,2′-bipyridine)dichlororuthenium(II) dihydrate (12 mM) in water and kept at 80 °C for 48 hours. To obtain the hydrogel base on iridium, the EFK-bpy peptide (12 mM) and dichlorotetrakis(2-(2-pyridinyl)phenyl)diiridium(III) (6 mM) was refluxed with acetonitrile, dried and dissolved in water. The europium based hydrogel was made by dissolved europium (II) chloride (4 mM) and EFK-bpy peptide (12 mM) in water and kept at 80 °C for 48 hours. All the products were dialyzed in water for 24 hours and freeze dried. The high-performance liquid chromatography (HPLC) analysis of the components in EFK-bpy-Eu, EFK-bpy-Ir and EFK-bpy-Ru hydrogels using a reverse-phase column (GE SOURCETM 5RPC ST 4.6/150) to investigate complexation degree was performed to confirm the reaction yield. As shown in Figure S11, the yield of EFK-bpy-Eu, EFK-bpy-Ir and EFK-bpy-Ru peptide were 57.61%, 25.44% and 15.54%, respectively.

### Preparation of hydrogels

For EFK-bpy, EFK-bpy-Ir hydrogel or EFK-bpy-Ru hydrogels, the peptide was dissolved in phosphate saline buffer (PBS, 10 mM, pH = 7.0) to the concentration of 12 mM. The peptide-PBS mixture was then thoroughly mixed by a vortex mixer (Scientific Instruments, USA) for 30 seconds at its maximum power and stored still for 1 h to hydrogelation. For EFK-bpy-Eu hydrogels, the EFK-bpy-Eu peptide was dissolved in phosphate saline buffer (PBS, 10 mM, pH = 7.0) to the concentration of 4 mM. For the WLFH, EFK-bpy-Eu, EFK-bpy-Ir and EFK-bpy-Ru were dissolved in phosphate saline buffer (PBS, 10 mM, pH = 7.0) to the concentration of 3.7, 0.56 and 0.28 mM, respectively. The mixture was thoroughly mixed by a vortex mixer for 30 seconds at its maximum power and the hydrogel was formed after the mixture was stored still for 1 hour. The concentration of the WLFH was 12 mM. The concentration of WLFH was 4 mM after three times of dilution.

### Mechanical Measurements

To study the mechanical property of these hydrogels, all hydrogels were prepared by these protocols mentioned above and transferred to the rheometer plate with care. The volume of each measurement was 50 μL. The measurement was undertaken in a Thermo Scientific Haake RheoStress 6000 using a frequency sweep mode with the frequency from 0.1 to 100 Hz at 0.1% strain. All these experiments were carried out at 20 °C and 0.50 mm gap. The geometry was 1°/20 mm of the cone.

### UV-Vis Spectroscopy and Fluorescence spectroscopy

All spectroscopy samples were prepared by diluting the hydrogel samples prepared above. The UV-Vis spectra were recorded by a JASCO FP-6500(JASCO Inc., Japan). The bandwidth was set as 0.2 nm. And fluorescence spectra were measured by a JASCO V-550(JASCO Inc., Japan) at different excitation wavelengths. Typically, 100 μL of hydrogels were suspended in 200 μL of PBS buffer and fractured by a vortex mixer (Scientific Instruments, USA) for 15 min at its maximum power.

### Circular dichroism (CD) spectra

All CD spectroscopy samples were measured in quartz cuvette with a path length of 1 mm. Typically, 20 μL of hydrogels were suspended in 980 μL of PBS buffer and fractured by a vortex mixer (Scientific Instruments, USA) for 15 min at its maximum power.

### Transmission Electron Microscope (TEM)

Transmission electron microscopic images were obtained using a JEM-200CX (JEOL Inc., Japan). The hydrogel was re-suspended in PBS (10 mM, pH = 7.0) 8 times of the original volume and mixed well. The sample solution was dropped on the Ultrathin Carbon Film on Copper and dried under the nitrogen.

### Preparation of printing pattern

All hydrogels were prepared by the protocols mentioned above. The concentration of EKF-Bpy-Eu dyrogels uesd was 4 mM while the concentration of other hydrogels were all 12 mM. The hydrogels were printed by an injector. Each spot was 20 μL. The pattern was printed on quartz glass to reduce the influence of the fluorescence from the glass.

## Electronic supplementary material

Supplementary Information
