# Supplementary material for: Printable Fluorescent Hydrogels Based on Self-Assembling Peptides
Source: Sci Rep. 2017 Aug 29;7:9691. doi: 10.1038/s41598-017-10162-y (PMC5574881; doi:10.1038/s41598-017-10162-y)
Supplement: Supplementary file 1 — Supplementary Information [file 41598_2017_10162_MOESM1_ESM.pdf]

Supplementary Information

Printable White-Fluorescent Hydrogels Based on Self-Assembling Peptides

Yifan Xia[1#], Bin Xue[1#*], Meng Qin[1], Yi Cao[1], Ying Li[2*] and Wei Wang[1*]

[1]National Laboratory of Solid State Microstructures, Department of Physics, Nanjing University, 22 Hankou Road, Nanjing, Jiangsu, 210093, P.R. China

[2]Collaborative Innovation Center of Atmospheric Environment and Equipment Technology, Jiangsu Engineering Technology Research Centre of Environmental Cleaning Materials, Jiangsu Key Laboratory of Atmospheric Environment Monitoring and Pollution Control, Jiangsu Joint Laboratory of Atmospheric Pollution Control, Jiangsu School of Environmental Science and Engineering, Nanjing University of Information Science & Technology, 219 Ningliu Road, Nanjing, Jiangsu, 210044, P.R. China

# These authors contributed equally to this work.

* Correspondence authors of this work.

Correspondence and requests for materials should be addressed to B. Xue (email: xuebinnju@163.com), Y. Li (yingliubc@gmail.com) or W. Wang (wangwei@nju.edu.cn)

Materials

4-Methyl-2,2'-bipyridine-4'-carboxylic acid-KFEFKFEF (EFK) are custom-synthesized from GL Biochem Ltd. Dichlorotetrakis(2-(2-pyridinyl)phenyl) diiridium(III), cis-bis(2,2'-bipyridine)dichlororuthenium(II) dihydrate, and europium(II) chloride are obtained from Sigma-Aldrich LLC or Tokyo chemical industry Ltd. All other chemicals are of chemical grade and purchased from Sinopharm Chemical Reagent Ltd. All materials are used without further purification if not mentioned.

HPLC analysis

HPLC analysis was performed on a ThermoScientific U3000 system at 280 nm with a GE SOURCETM 5RPC ST 4.6/150 column. The mobile phases are solvent A: 0.1% TFA in H_2O and solvent B: 0.1% TFA in CH_3CN with gradient: 0 to 2 minute, 5% A, 2 to 5 minute, 5% A to 20% A, 5 to 10 minute, 20 % to 30 % A, 10 to 15 minute, 30% to 50% A, 15 to 18 minute, 50% to 100%, 18 to 21 minute, 100%, 21 to 24 minute, 100% to 5% A, 24 to 25 minute, 5% A. All samples were dissolved in methanol for injection. Each fraction was collected manually to determine the component by fluorescence spectroscopy.

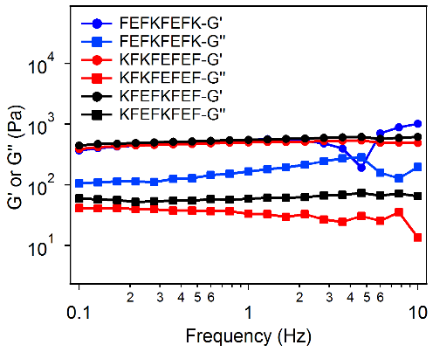

Figure S1 The mechanical properties of hydrogels formed by peptides with different sequences (FEFKFEFK, KFKFEFEF, and KFEFKFEF) at the concentration of 12 mM.

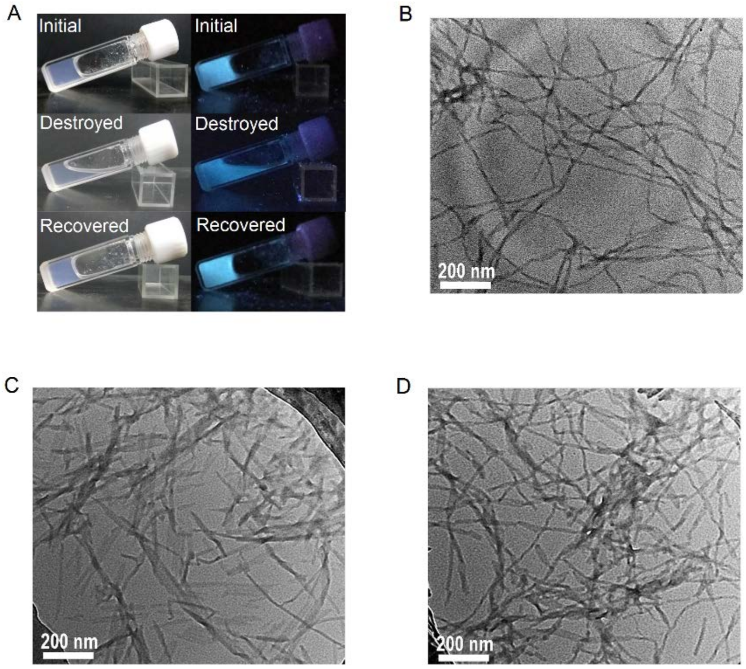

Figure S2 (A) The optical images of the initial, ultrasound-destroyed, and recovered EFK-bpu-Eu hydrogels (4 mM) under the natural light (left) and UV1 (right). (B-D) TEM images of the fibrous networks of (B) initial, (C) ultrasound-destroyed, and (D) recovered EFK-bpy-Eu hydrogels.

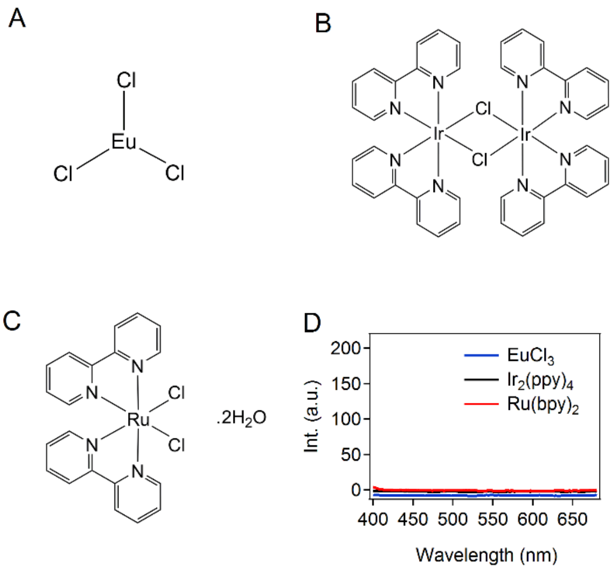

Figure S3 The chemical structural of the three different transition metal ionic compounds used to form ionic complexes. (A) Europium (III) chloride, (B) Dichlorotetrakis (2-(2-pyridinyl)phenyl)diiridium(III), and (C) cis-bis(2,2′-bipyridine)dichlororuthenium(II) hydrate (D) The fluorescence emission spectrum of the Ir, Eu and Ru compounds shown in A-C (λ_{ex}=365 nm, 4 mM).

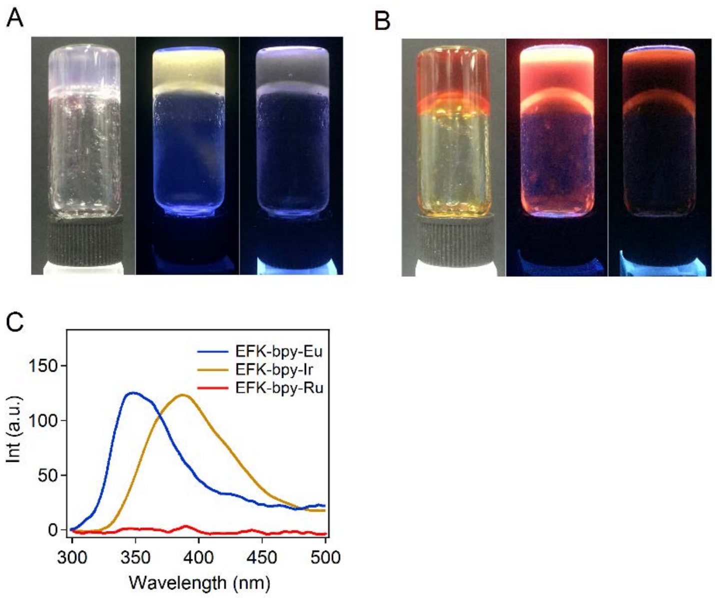

Figure S4 The optical images of (A) EFK-bpy-Ir gels (12 mM), and (B) EFK-bpy-Ru gels (12 mM) under natural light (left), UV_1 (middle), and UV_2 (right). (C) The fluorescence emission spectrum of the EFK-bpy-Eu (λ_{ex}=254 nm; 1.33 mM), EFK-bpy-Ir (λ_{ex}=254 nm; 4 mM), and EFK-bpy-Ru (λ_{ex}=254 nm; 4 mM).

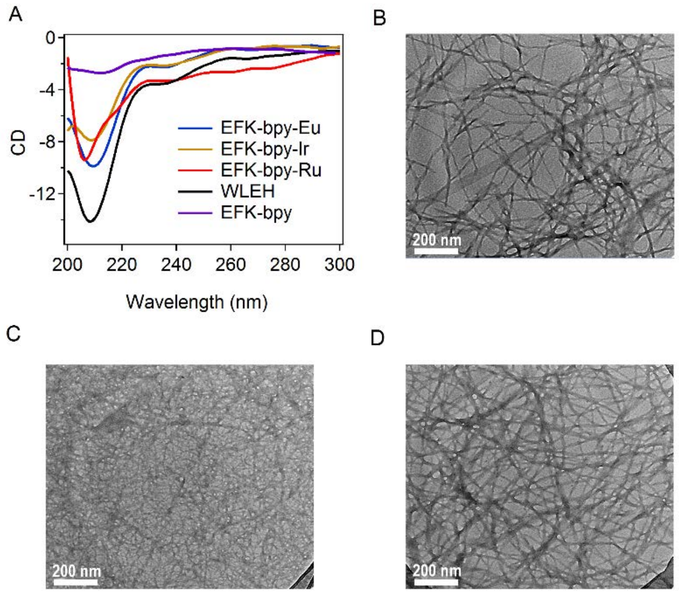

Figure S5 (A) The CD spectra of the EFK-bpy hydrogel (0.24 mM), EFK-bpy-Eu hydrogel (0.08 mM), EFK-bpy-Ir hydrogel (0.24 mM), EFK-bpy-Ru hydrogel (0.24 mM), and White Light Fluorescent Hydrogel (WLFH) (0.24 mM). TEM images of the fibrous networks of (B) EFK-bpy-Ir hydrogel, (C) EFK-bpy-Ru hydrogel, and (D) WLFH.

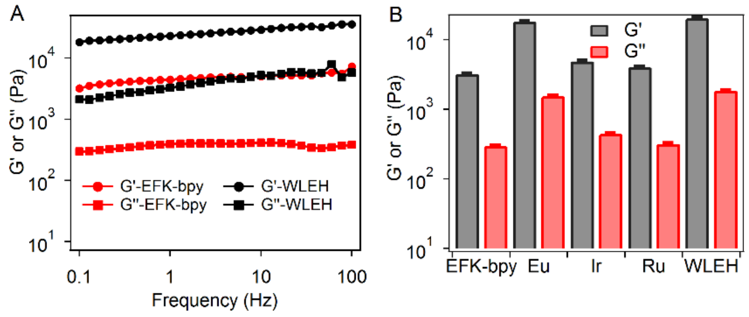

Figure S6 The Mechanical properties of the hydrogels. (A) The rheological properties of the gels formed by EFK-bpy peptide (12 mM) and the WLFH (12 mM) at 0.1 % strain in the frequency range of 0~100 Hz. (B) The G' and G'' of the hydrogels subject to EFK-bpy (12 mM), EFK-bpy-Eu (4 mM), EFK-bpy-Ir (12 mM), EFK-bpy-Ru (12 mM), and WLFH (12 mM) at 1 Hz and 0.1% strain.

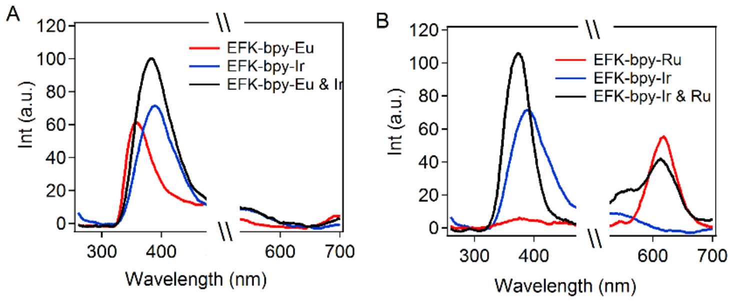

Figure S7 (A) The emission spectrum of the EFK-bpy-Eu (λ_{ex}=254 nm; 1.33 mM), EFK-bpy-Ir (λ_{ex}=254 nm; 4 mM) and EFK-bpy-Eu-Ir co-assembly hydrogels. The concentrations of EFK-bpy-Eu and EFK-bpy-Ir in the mixture were 0.66 mM and 2 mM, respectively. (B) The emission spectrum of the EFK-bpy-Ru (λ_{ex}=254 nm; 4 mM), EFK-bpy-Ir (λ_{ex}=254 nm; 4 mM) and EFK-bpy-Ru-Ir co-assembly hydrogels. The concentrations of EFK-bpy-Ir and EFK-bpy-Ru in the mixture were 2 mM and 2 mM, respectively.

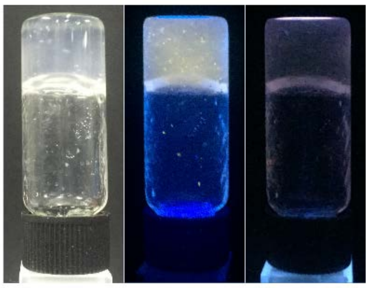

Figure S8 The optical images of WLFH (12 mM) under natural light (left), UV_1 (middle) and UV_2 (right).

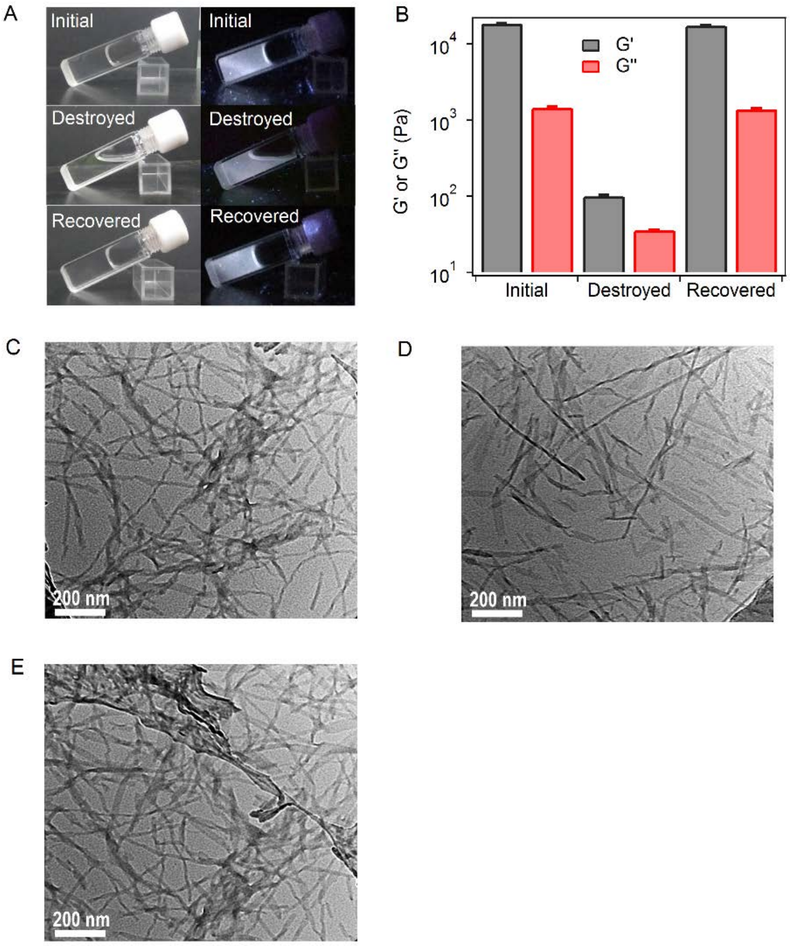

Figure S9 (A) The optical images of the initial, ultrasound-destroyed, and recovered WLFH (12 mM) under the natural light (left) and UV_1 (right). (B) The G' and G'' of WLFH. (C-E) TEM images of the fibrous networks of (C) initial, (D) ultrasound-destroyed, and (E) recovered WLFH.

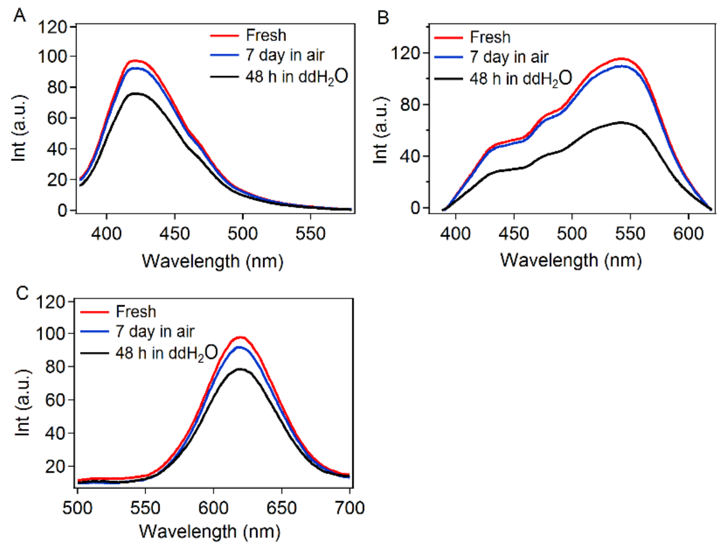

Figure S10 The photostability of the fluoresent hydrogels. (A) The emission spectrum of the EFK-bpy-Eu (λ_{ex}=365 nm; 1.33 mM) stored under different conditions. (B) The emission spectrum of the EFK-bpy-Ir (λ_{ex}=365 nm; 4 mM) stored under different conditions. (C) The emission spectrum of the EFK-bpy-Ru (λ_{ex}=365 nm; 4 mM) stored under different conditions.

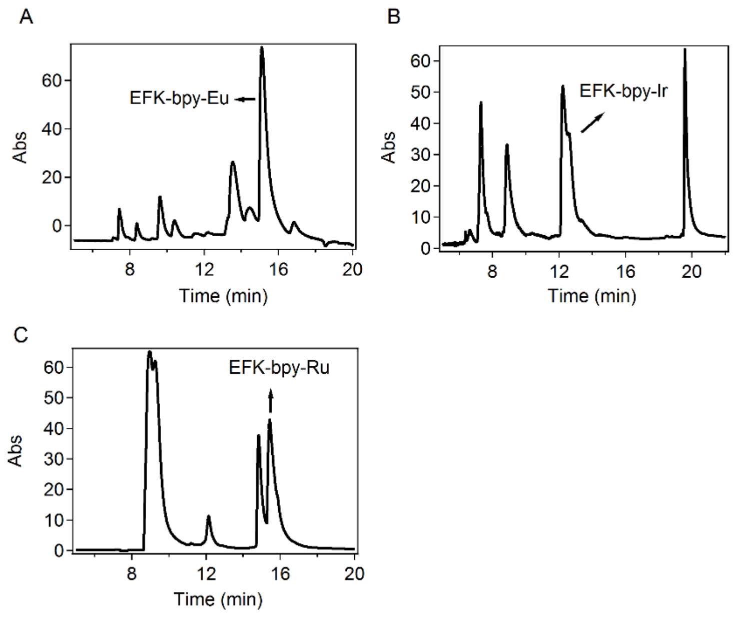

Figure S11 The HPLC traces of (A) EFK-bpy-Eu hydrogel, (B) EFK-bpy-Ir hydrogel and (C) EFK-bpy-Ru hydrogel at 280 nm.
